# Epstein–Barr Virus Epithelial Cancers—A Comprehensive Understanding to Drive Novel Therapies

**DOI:** 10.3389/fimmu.2021.734293

**Published:** 2021-12-10

**Authors:** Shuting Han, Joshua K. Tay, Celestine Jia Ling Loh, Axel Jun Ming Chu, Joe Poh Sheng Yeong, Chwee Ming Lim, Han Chong Toh

**Affiliations:** ^1^ Division of Medical Oncology, National Cancer Centre Singapore, Singapore, Singapore; ^2^ Department of Otolaryngology—Head & Neck Surgery, National University of Singapore, Singapore, Singapore; ^3^ Duke-NUS Medical School, Singapore, Singapore; ^4^ Department of Anatomical Pathology, Singapore General Hospital, Singapore, Singapore; ^5^ Department of Otolaryngology—Head & Neck Surgery, Singapore General Hospital, Singapore, Singapore

**Keywords:** Epstein–Barr virus, tumour microenvironment, nasopharyngeal cancer, gastric cancer, lymphoepithelioma-like carcinoma

## Abstract

Epstein–Barr virus (EBV) is a ubiquitous oncovirus associated with specific epithelial and lymphoid cancers. Among the epithelial cancers, nasopharyngeal carcinoma (NPC), lymphoepithelioma-like carcinoma (LELC), and EBV-associated gastric cancers (EBVaGC) are the most common. The role of EBV in the pathogenesis of NPC and in the modulation of its tumour immune microenvironment (TIME) has been increasingly well described. Much less is known about the pathogenesis and tumour–microenvironment interactions in other EBV-associated epithelial cancers. Despite the expression of EBV-related viral oncoproteins and a generally immune-inflamed cancer subtype, EBV-associated epithelial cancers have limited systemic therapeutic options beyond conventional chemotherapy. Immune checkpoint inhibitors are effective only in a minority of these patients and even less efficacious with molecular targeting drugs. Here, we examine the key similarities and differences of NPC, LELC, and EBVaGC and comprehensively describe the clinical, pathological, and molecular characteristics of these cancers. A deeper comparative understanding of these EBV-driven cancers can potentially uncover targets in the tumour, TIME, and stroma, which may guide future drug development and cast light on resistance to immunotherapy.

## Introduction

Epstein–Barr virus (EBV) is a ubiquitous oncovirus affecting more than 90% of adult populations globally and has been classified as a type I carcinogen leading to 1.5% of all cancers (1). Latent EBV infection is known to be associated with multiple lymphoid malignancies and epithelial cancers. Common EBV-associated epithelial cancers include nasopharyngeal carcinoma (NPC), lymphoepithelioma-like carcinoma (LELC) and EBV-associated gastric cancers (EBVaGC). Rarely, EBV-positive breast cancer, thyroid cancer, salivary gland cancers, and hepatobiliary cancers have been reported. The pathogenesis and somatic mutational landscapes of NPC and EBVaGCs have been described. However, significantly less is known about LELC owing to the rarity of the disease. Interestingly, although they may differ in clinical presentations and anatomical site, what these viral-driven cancers have in common is a characteristic immune-suppressed tumour immune microenvironment (TIME) driven primarily by EBV. The similarities in the somatic mutational landscape between NPC and LELC also highlight the influence of the transforming virus on epithelial cells despite the different anatomical locations. Hence, a deeper understanding of these similar yet distinct EBV-driven epithelial cancers may guide future diagnostic and therapeutic strategies.

## Epidemiology of EBV-Driven Epithelial Cancers

NPC is a head and neck cancer with a median age of onset between 50 and 60, with men two to three times more likely to be affected compared to women. NPC is extremely uncommon in the Western hemisphere with an incidence of 0.5 to 2 per 100,000 person years (2). In contrast, NPC is endemic in Southern China, Hong Kong, and parts of Southeast Asia, with incidence rates greater than 30 per 100,000 person years in high-risk populations. Other endemic regions include North Africa and the Middle East (3). First-degree family members of NPC patients have been observed to have a markedly elevated risk of NPC (4). Additionally, persons migrating from high-risk areas continue to retain an increased risk for the disease, with this risk diminishing with each successive generation (3). A hypothesis of early human migration pattern leading to specific endemic regions of NPC in Africa, Middle East, and China has also been proposed (5).

LELC, unlike NPC, has been observed to arise from many organs including the lung, breast, thyroid, salivary gland, liver, and prostate. Similar to NPC, LELC had been reported to be more common in Asians, especially persons of Southern Chinese and Southeast Asian descent. In the reported pulmonary LELC series, the median age of onset tends to be younger than that of NPC and more non-smokers are noted (6, 7). Gender predilection is not established in pulmonary LELC (PLELC) although a few series show a higher proportion of females (8, 9). Other descriptions of LELC include LEL-intrahepatic cholangiocarcinoma and LEL-hepatocellular carcinoma, which appear to be associated with chronic hepatitis B and C infections rather than EBV.

EBVaGC is a subset of gastric cancer but with distinct clinical and pathological characteristics. Like NPC, EBVaGC is more predominant in men and in younger individuals (10). However, the proportion of EBVaGC among cases of gastric cancer is lower in Asia (2%–10%) compared to Western countries such as Germany and the United States (15%–18%) (11).

## Etiology of EBV-Driven Epithelial Cancers

Environmental risk factors for NPC include consumption of salt-cured, preserved, or fermented foods containing nitrosamine or aflatoxin, and exposure to radiation (12, 13). However, EBV infection is thought to be critical to the etiology of NPC (14). This is supported by the detection of EBV in dysplastic nasopharyngeal epithelium, as well as the clonal nature of EBV identified in NPC biopsies, suggesting that EBV infection is an early event in carcinogenesis (15). In addition, elevated serum EBV capsid antigen (VCA) Immunoglobulin A (IgA) or EBV DNA titers are associated with an increased risk of developing NPC, with raised levels associated with advanced disease and observed to precede the clinical onset of NPC (4, 16). Certain HLA haplotypes (A2, B17, and BW46) and other susceptible gene loci also may predispose one to NPC (17). A prevailing hypothesis suggests that early EBV infection can lead to latency and EBV-associated epithelial cancers such as NPC, while infection in adolescence or adulthood leads to infectious mononucleosis and also may result in EBV-associated lymphoid cancers such as Hodgkin’s lymphoma (18).

Although the etiology of the LELC is unclear, it also has a strong association with EBV. Begin et al. first detected EBV in LELC tissue obtained from a 40-year-old non-smoking woman of Southeast Asian descent (19). Since then, there has been accumulating evidence showing the presence of EBV-encoded RNA (EBER) in almost all LELC tumours (20).

Likewise, EBER is also expressed in EBVaGC cells, supporting the role of EBV in the pathogenesis of the disease (21). Other risk factors include male gender, an increased intake of salty food, and exposure to wood dust or iron fillings, in comparison to EBV-negative gastric cancer (22).

## Histomorphology and Immunohistochemistry

**Figure 1:**
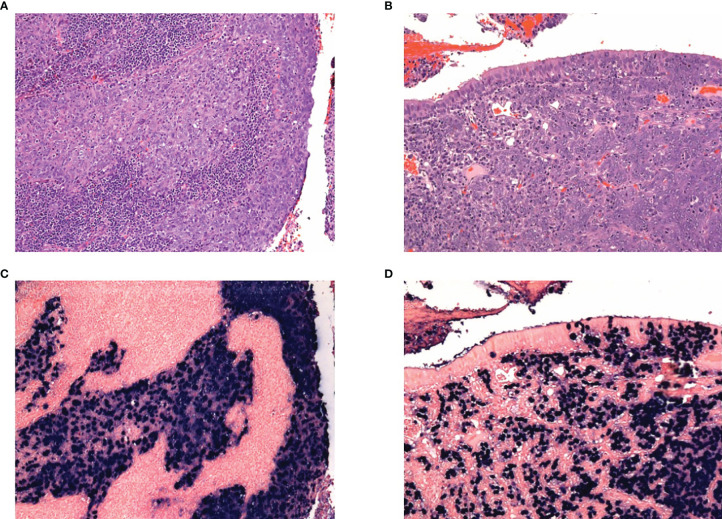
H&E of nasopharyngeal cancer showing tumours with **(A)** Regaud appearance, showing distinct islands of tumour cells, and **(B)** Schmincke appearance, with interdigiting tumour cells and inflammatory infiltrate. Corresponding EBER-ISH for the Regaud and Schmincke patterns in **(C)** and **(D)** respectively.

NPC has been classified into two main histological types by the World Health Organisation (WHO)—squamous cell carcinoma (type I) and undifferentiated carcinoma (types IIa and IIb). The latter, which is associated with EBV, can be further divided into the keratinising form (type IIa), and non-keratinising form (type IIb) (23). Type IIa NPC tumours have a stratified arrangement with fairly well-defined margins. Type IIb NPC tumours are the most common, with cellular features of round vesicular nuclei with prominent nucleoli, indistinct margins and a syncytial appearance. Histologically, type IIb tumour epithelial cells may appear as well-defined areas distinct from the tumour microenvironment (Regaud appearance), or in loosely connected cells admixed with the tumour microenvironment (Schmincke appearance) (24). The protein expression of LMP1 and 2A/B is variable in type IIb NPC tissues (25) (**Figure 1**).

**Figure 2:**
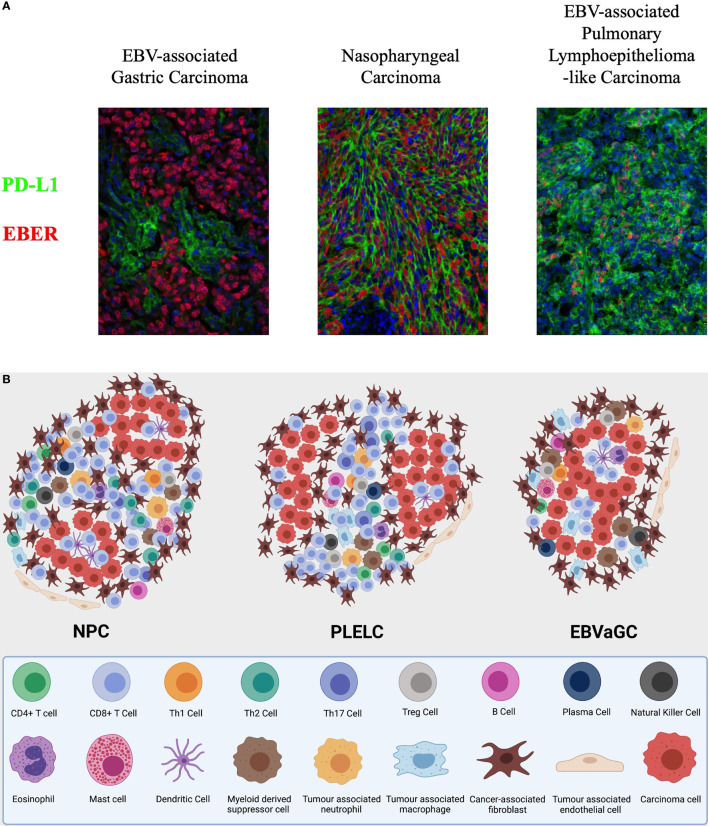
**(A)** Integrated multispectral imaging technique that simultaneously detects EBER and immune cell protein marker (PDL1) in formalin-fixed, paraffin-embedded (FFPE) tissues of EBV-associated epithelial cancers—nasopharyngeal carcinoma (NPC), pulmonary lympho-epithelioma-like carcinoma (PLELC), and EBV-associated gastric cancer (EBVaGC). **(B)** A symbolic illustration of the tumour immune microenvironment of nasopharyngeal carcinoma (NPC), lymphoepithelioma-like carcinoma (LELC), and EBV-associated gastric carcinoma (EBVaGC). NPC and LELC are similar with an abundance of immune cells including predominantly T cells. The cell types present include CD4+ T cells, CD8+ T cells, T regulatory cells, B cells, NK cells, myeloid cells, and fibroblasts in the stroma and some immune cells infiltrating the tumour nests. There are more CD8 than CD4 T cells but the illustration does not represent relative abundance. The fibroblasts are thought to be higher around the edge of the tumour nests. Immunosuppressive cells including Tregs, tumour associated macrophages, and tumour associated neutrophils are present. Inspired by Tan GW, Visser L, Tan LP, van den Berg A, Diepstra A. The Microenvironment in Epstein-Barr Virus-Associated Malignancies. *Pathogens*. 2018;7(2):40. doi:10.3390/pathogens7020040.

Most LELC tumour cells are large undifferentiated cells with vesicular nuclei, growing in a syncytial trabecular pattern, accompanied by a heavy lymphoid stromal infiltration (9, 26–31) (**Figures 2A, B**). This is strikingly similar to the histological pattern of Type IIb NPC as described above. However, there are some types of LELC that display a slightly different histological pattern and they are described below:

Lymphoepithelioma-like-intrahepatic cholangiocarcinoma (LEL-ICC) tumour cells are poorly differentiated and consist of sheets of neoplastic cells, poorly formed glands, surrounded and accompanied by a dense lymphocytic stroma infiltration (29, 32). Tumour cells are large, with prominent nucleoli, abundant syncytial cytoplasm and vesicular chromatin (29). LEL-ICC can range from a well-differentiated glandular pattern to a syncytial pattern with undifferentiated cells. Thymic LELC, on the other hand, has tumour cells arranged in nest-like patterns or stripe-shaped in collagen fibrous interstitial tissue containing lymphocytes. They have large nuclei with irregular chromatin and mitosis. However, in the thymus, some LELC can lack prominent lymphoid stroma and can resemble non-keratinising squamous cell carcinoma (27).

Interestingly, a recent study on PLELC also demonstrated that they have a morphologically continuous spectrum, ranging from classic poorly differentiated tumours with intense lymphocytic infiltration to non-classical morphology with little lymphocytic infiltration (27). A more recent study by the same group showed that the EBV-associated pulmonary carcinoma with non-classical morphology and low lymphocytic yield also had similar molecular characteristics as classic LELC. This again demonstrates a broader category of EBV-associated carcinomas with some not demonstrating the classical pattern of lymphocyte infiltration.

EBVaGC cells are poorly differentiated adenocarcinoma with varying amount of infiltrating lymphoid stroma. In the early stage, EBVaGC has a “lace-like” pattern, with irregularly anastomosing tubules and cords. A vast majority of EBV-associated epithelial cancers have positive EBER-ISH staining on light microscopy and is considered part of the diagnostic criteria for LELC and EBVaGC. Likewise, all the above epithelial tumours stain positively for epithelial markers, though studies suggest CK7 expression may be reduced compared to EBV-negative GCs (33) (**Table 1**).

**Table 1:** A table summarising the clinical, epidemiologic, etiologic, histological and molecular features of nasopharyngeal carcinomas (NPCs), lymphoepithelioma-like carcinomas (LELCs) and EBV-associated gastric carcinomas (EBVaGCs).

	NPC	LELC	EBVaGC
**Etiology**	EBV infection Other risk factors: smoking, food containing nitrosamine and aflatoxin, exposure to radiation	EBV infection	EBV infection
**Epidemiology **	Rare in the West and endemic in Southern China, Hong Kong, Southeast Asia, North Africa, and Middle East Male > Female	More common in Asians, especially persons of South China and Southeast Asian descent Female > Male	Lower frequency of occurrence in Asian countries compared to Western countries 2-10% of gastric cancers Male > Female
**Clinical features **	Presence of painless neck lump, blocked ear sensation, tinnitus, blood stains in mucus or saliva headache, diplopia, facial numbness due to cranial nerve involvement at skull base Organs most frequently involved in distant metastases are bones, lungs, liver	Cough with blood-tinged sputum Other symptoms include: Chest pain, loss of weight and/or appetite, intermittent fever and chills, night sweats, joint pain, and exertional dyspnea	Loss of weight, nausea, early satiety, epigastric pain, dysphagia, gastrointestinal bleed, iron deficiency anaemia Common sites of metastasis include liver, peritoneal surfaces, and distant lymph nodes
**Histomorphology and immunohistochemistry**	Squamous cell carcinoma (type I) and undifferentiated carcinoma (type IIa and IIb) Type IIa (keratinizing form) presents with round vesicular nuclei with prominent nucleoli, indistinct cell margins, with a syncytial appearance Type IIb (non-keratinizing form) can be found in irregular and moderately well-defined areas, or in loosely connected cells admixed with the tumour microenvironment Type 2b: EBER-ISH positive	Large undifferentiated cells with vesicular nuclei, growing in a syncytial trabecular pattern, accompanied by a variable lymphoid stromal infiltration Similar to the histological pattern of Type IIb NPC	Poorly differentiated adenocarcinoma with varying amount of infiltrating lymphoid stroma Positive EBER-ISH
**Key molecular and immune characteristics**	NF-kB pathway activation through LMP1, silencing of CDKN2A, CCND1 amplification, JAK/STAT, NOTCH, PI3K pathway, TP53 mutations Immune cell signaling esp. IFN-related genes, MHC Class I downregulation or mutation Chromosomal instability phenotype EBV-CIMP (CpG island methylator phenotype)	NF-kB pathway activation, silencing of CDKN2A, CCND1 amplification, TP53 mutations, JAK/STAT, NOTCH Immune cell signaling esp. IFN-related genes Chromosomal instability phenotype	Mutations in PIK3CA, overexpression of PD-L1/2, silencing of CDKN2A and immune cell signaling EBV-CIMP

## The Role of EBV in Pathogenesis of EBV-Associated Epithelial Cancers

EBV, also known as human herpes virus type 4, is a 172-KB double-stranded DNA (dsDNA) virus containing expression product of 80 proteins and 46 functional small-untranslated RNA. EBV exhibits dual tropism—infecting both lymphoid and epithelial cells, though the virus is hardly detected in healthy epithelium. While it is transmitted through saliva, the site of persistence is thought to be the resting memory B cells in the peripheral blood, though others suggest the site of persistence to be in the salivary gland, bone marrow, or the resident plasma cells in the nasopharynx (34). In terminally differentiated plasma cells, the lytic virus life cycle is reactivated (35). Infection of nasopharyngeal mucosal tissue likely occurs as a result of viral particles released during lytic replication (36–38) and entry into epithelial cells *via* Ephrin receptor A2 (EphA2) (39). EBV genome and viral latent gene products in invasive carcinoma and dysplastic lesions suggest that the EBV latent infection precedes clonal expansion of tumour cells and invasive growth (40).

During latency, EBV genome exists as extrachromosomal episomes in the nucleus and expresses some proteins. NPC displays Type II Latency Programme genes—Latent Membrane Protein 1 (LMP1), LMP2A/B, Epstein–Barr Nuclear Antigen (EBNA)1, BamHI-A rightward frame 1 (BARF1), EBER1 and 2, BamHI-A rightward transcripts (BARTs), and microRNA of BARTs (miR-BARTs), all of which are implicated in tumourigenesis. LMP1, found *in vitro* to be essential for B-cell transformation and immortality, is thought to be an early and key oncogenic driver of NPC. It is present in 20%–60% of NPC tumours by immunohistochemistry (IHC) but is virtually present in all pre-malignant or pre-invasive lesions (25). LMP1 is considered to be a constitutively active homologue of the CD40 receptor (a member of the TNF receptor superfamily) by activation of nuclear factor kappa-light-chain-enhancer of activated B cells (NF-kB) pathway (40), as well as multiple signaling pathways including mitogen-activated protein kinase (MAPK), phosphatidylinositol 3-kinase (PI3K), and signal transducer and activator of transcription (STAT). LMP1 activates NF-κB *via* two C-terminal activation regions (CTAR1 and CTAR2). CTAR2 activates canonical NF-κB through TRAF6/TAK1/IKKβ, while CTAR1 recruits TRAF 1, 2, 3, and 5 and activates non-canonical NF-κB in an NIK/IKKα-dependent manner (41–44). LMP1 also promotes stem-like properties in NPC cell lines (45) and also has anti-apoptotic properties (46), resulting in tumour advantage and survival. Additionally, LMP1 is implicated in metabolic reprogramming through the de-regulation of several glycolysis enzyme including hexokinase 2 (HK2) (47) and glucose transporter 1 (GLUT-1) signalling cascade (48). It is also implicated in angiogenesis *via* predominantly vascular endothelial growth factor (VEGF) upregulation, and metastasis and epithelial–mesenchymal transition (EMT) through the matrix metalloproteinase-2 (MMP2), MMP-9, and microRNA (miRNA)-200 downregulation of E-cadherin (49). The multiple roles of LMP1 in modulating the inflamed yet immune-suppressed tumour immune microenvironment and enabling tumour to evade host immune cells will be discussed further in the subsequent sections (25).

LMP2A, a B-cell receptor (BCR) homologue, also has multiple oncogenic properties through the modulation of various oncogenes and tumour suppressor genes (TSGs). It can promote proliferation, angiogenesis, cell invasion, and metastasis, with anti-apoptotic effects as well (50). EBNA1 is known for persistent maintenance of EBV genome in NPC cells by governing replication and mitotic segregation of EBV episome, and also causes genomic instability and promotes tumour cell survival (51). BARTs are expressed highly in EBV-transformed epithelial cancers while low in lymphoid cancers (52), and include alternatively spliced transcripts containing long non-coding RNAs (lncRNAs) and miR-BARTs, both of which may also be related to NF-kB activation and tumour proliferation. miR-BARTs are short single-stranded RNA molecules that regulate gene expression post-transcriptionally and have also been found to be relevant in anti-apoptosis, metastasis, and immune evasion (53). They regulate the expression of LMP1 and various host genes (i.e., miR-BART7 and miR-BART9 target PTEN; miR-BART3 targets tumour suppressor gene DICE1), overall influencing multiple pathways to promote tumour proliferation and metastases (54, 55) BART lncRNA has also recently been shown to be related to host cell expression of genes involved in cell adhesion, inflammation, and may also play a role in epigenetic modulation (56). BARF1 is a viral oncoprotein expressed in latently infected EBV-positive epithelial tumour cells. It is a homologue of the human c-fms protein which is the receptor for human colony-stimulating factor 1 (hCSF1). Hence, BARF1 may regulate the proliferation and differentiation of mononuclear cells to macrophages and may also stimulate the secretion of IFN-α (57).

NPC is the best-studied EBV-associated epithelial cancer. It has been proposed that loss of heterozygosity (LOH) of 3p and 9p is an early event in NPC pathogenesis (58, 59), possibly due to environmental triggers. The loss of tumour suppressor genes at these loci result in low-grade, pre-invasive lesions that become susceptible to EBV infection, which facilitates the acquisition of additional genomic aberrations (25). Hypermethylation of RASSF1, a tumour suppressor gene at 3p21.3, may also be a key event in tumourigenesis (60). CDKN2A/p16 (9p21) homozygous deletion or overexpression of cyclin D1 overcomes EBV-induced senescence and allows stable EBV infection in cell lines, further supporting these molecular alterations as early events. Disruption in TP53 pathways and G1/S cell cycle checkpoint in premalignant nasopharyngeal lesions may be another important event for EBV persistence and tumour initiation (61–63).

There has been limited research into the pathogenesis of LELC so far. Recent genomic studies have revealed a similar mutational landscape to NPC, perhaps suggesting a similar pathogenesis through NF-kB signaling. LMP1 is also highly expressed in 20% of LELC tumours (64). The distribution of LELC at different organ sites, yet leading to a similar poorly differentiated disease phenotype, points to the complex nature of EBV infection. Whether LELC arises from columnar, squamous, or glandular epithelium, or other specialised cell types remains a question to be answered. Recent data also suggest that miR-BARTs (BART5-3P and BART 20-3P) may be involved in tumourigenesis of PLELC (65). The type of EBV latency has also not been fully explored in LELC although LMP1 and LMP2 have both been found to be expressed, suggesting a type II or III EBV Latency Programme (66).

In EBVaGC, it is postulated that the virus present in saliva is ingested and infects the epithelial cells of stomach lining directly, or that during the lytic phase (re-activation), resident B lymphocytes in stomach mucosal tissue release EBV to infect epithelial cells. Ephrin receptor A2 as well as integrins and non-muscle myosin heavy chain IIA (NMHCIIA) serve as cofactors and play an important role in EBV epithelial cell entry (39). It was also proposed that EBV-infected lymphocytes contact epithelial cells *via* integrin β1/β2, with upregulation of adhesion molecule-1 for increased cell–cell contact. The viral particle is then transmitted by clathrin-mediated endocytosis pathway and establishes latent infection in gastric epithelial cells (39). Akin to NPC, it has been observed that EBV-positive gastric tumour cells are clonal and EBV was not generally detected in normal stromal cells, metaplasia, gastric mucosa, and lymphocytes. It is postulated that EBV infection again is an early event and occurs during the dysplastic phase of tumour progression. EBV anti-VCA and anti-EBNA antibody titers are higher in persons with dysplasia on gastric biopsy, suggesting that EBV reactivation could be related to the early pathogenesis of gastric carcinoma (67).

In contrast to NPC and LELC, EBVaGCs exhibit Type I latency, with the expression of BARTs, low levels of EBNA1, and lack of EBNA2 and LMP1 (68). Less commonly, LMP2A is expressed in approximately 40% of EBVaGCs (69, 70). Genes associated with EBV lytic replication have also been observed in EBVaGCs (71); however, the role that they play remains uncertain. After infection with EBV, or transfection with EBV genes (BARF0, EBNA1, and LMP2A), gastric cancer cell lines downregulate miR-200 transcripts, leading to reduced E-cadherin expression, which is thought to be an important step in EBVaGC carcinogenesis (72).

## Somatic Molecular Mutational Landscape

The somatic mutational landscape of NPC reveals a complex interplay of viral genes, acquired genetic and epigenetic changes during the clonal expansion of EBV-infected nasopharyngeal epithelial cells (**Table 2**). Massive parallel sequencing technology has allowed better characterisation of the mutational landscape of EBV-driven cancers. Traditionally, NPC is seen as a homogenous tumour with a relatively low mutational rate reported in earlier studies. However, a recent study revealed a higher somatic mutational rate (median > 50 mutations/tumour) in a panel of 111 micro-dissected EBV-positive NPC tumour specimens (73, 74), suggesting a more heterogenous mutational landscape than previously imagined and a mutation rate more similar to other solid organ cancers. Compared to HPV-driven cancers, integration of EBV into the host genome is uncommon and has been reported in 9.6% of NPC tumours, at sites near to tumour suppressor and inflammation related genes (75).

**Table 2:** Genomic and epigenomic differences between nasopharyngeal carcinomas (NPCs), lymphoepithelioma-like carcinomas (LELCs) and EBV-associated gastric carcinomas (EBVaGCs).

,	NPC	LELC	EBVaGC
EBV related Latency genes	• Type II Latency genes: LMP1, LMP2A/B, EBNA1, BARF1, EBER1 and 2, BARTs and miR-BARTs	• Unclear	• Type I Latency genes: BARTs and miR-BARTs. BARF1, low levels of EBNA1, LMP2
Key somatic mutations	• TP53 mutation • NF-kB • NF-kB negative regulators (TRAF3, CYLD, ) • Cell cycle genes: CCDN1 amplifications • Mutations in PI3K pathway genes (PIK3CA, PTEN, ERBB3, BRAF1, NF1, FGFR2, FGFR3) • MAPK pathway genes • HLA Class I, NLRC5	• NF-kB, in particular NF-kB1A • JAK/STAT Cell cycle genes: CCND1 amplifications • TP53 mutation • PIK3CA amplification • NF-kB pathway genes: FADD, TRAF2, TRAF6, and CARD11 • Bcl-2 upregulation • Amplification of MDM2, and DAXX • Deletion of CDKN2A, CDKN2B, MTAP, RB1 and type I IFN genes	• Mutation in BCOR and amplification of 9p24.1 • Downregulation of TSG (TFF2, RBP4, HOXA9, LRRN1, RAP1GAP) • Upregulation of oncogenes (CDH17, CDX1, ETV4, PPP1R1B) • Alterations of hedgehog inhibitor genes and Wnt pathways • PIK3CA and ARIDA11 amplifications • Other common mutations: PTEN, SMAD4, CTNNB1 and NOTCH1
Epigenetic signatures	• Global CpG hypermethylation, EBV-CpG island methylator phenotype (CIMP) • DNA methyltransferases (DNMTs) • Hypermethylation in the tumour suppressor genes (TSGs) including RASSF1, CDKN2A, CDH1 and PTEN etc • DNA methylation changes are frequently reported in 3p21.3, 9p21, and 6p21.3 regions • Methylation of other TSG: ADAMTS9, PTPRG, ZMYND10, FBLN2, CRYAB, CADM1, THY1, MMP19, DUSP6, MIPOL1, and LTBP2 • Mutations in epigenetic modulators (ARID1A, BAP1, KMT2B, KMT2C, KMT2D, TSHZ3, HDAC4, PAXIP1) • Epigenetic silencing of CpG demethylase (TET1), mitotic checkpoint regulator (CHFR), cadherins (CDH1, CDH13), matrix metalloproteinase (MMP19), transcription factor (HOPX) etc • H3K27me3 elevation	Unknown	• Global CpG hypermethylation, EBV-CpG island methylator phenotype (CIMP) • STAT3 phosphorylation and increased DNMT1 level • TET2 downregulation by BARF0 and LMP2 • Silencing of CDKN2A • Methylation of tumour suppressor genes (APC, PTEN, and RASSF1a etc), cell adhesion molecules (THBS1 and E-cadherin) and others including ACSS1, FAM3B, IHH and TRABD • Reduction of H3K27me3 around TSS • Global reduction of H3K27me3, H3K9me3, and H4K20me3
Chromosomal instability	• Copy number gains in chr 1q, 2q, 3q, 3q, 6q, 7q, 8p, 8q, 11q, 12p, 12q and 17q • Cyclin D1 (located at 11q13) highest copy number change • MYC (8q24), ERBB-PI3K pathway, TBR (12p13), TERC (3q26.3), and ESR (6q25) copy number gains • Losses of chr 1p, 3p, 9p, 9q, 11q, 13q, 14q, and 16q • Deletions in negative regulators of NF-kB pathway (TRAF3, CYLD, NFKBIA, and NLRC5)	• Copy number gains in chr 5p, 12p and 12q • Copy number losses in chr 3p, 5q, 13q, 14q, and 16q • Significantly amplified chromosome regions include 7p11.2, 9p24.1, 11q13.3, 12p13.2 • Significantly deleted regions include 3p21.31, 3p25.3, 5q14.1, 9p21.3, 11q23.3, 13q14.2, 14q32.32, and 17p13	• Amplifications in chr 3q, 7, 9p, and 20 • Deletions in chr 18q
Key pathways	• NF-kB pathway, JAK/STAT, RAS/RAF/ERK/MAPK, PI3K/Akt, JNK/SAPK, NOTCH, TGF-beta/activin A,	• NF-kB, JAK/STAT pathway, PI3K/Akt	• STAT3, PI3K/Akt, Wnt pathway
Others	• Metabolic re-programming • Cadherin members (FAT1, FAT2, FAT3) • Autophagy-related genes (ATG2A, ATG7, ATG13)	NA	NA

Li et al. identified key mutational signatures in NPC tumours based on whole exome sequencing (WES), including the deamination of 5-methyl-cytosine, defective DNA mismatch repair, and an APOBEC/AID signature, which is related to innate immune response against viral infections (74). Common somatic mutations in NPC include TP53 and genes in the NF-kB pathway, especially TRAF3 and CYLD (74). Other genomic aberrations include MAPK pathway genes (NRAS, FGFR2, FGFR3, BRAF1, NF1, and ERBB3), PI3K pathway genes (PIK3CA and PTEN), and the major histocompatibility complex (MHC) class I gene HLA-A (74). A recent whole genome sequencing (WGS) paper by the same group revealed a similar set of mutational genes, including that of innate immunity, adaptive immunity, and immune escape mechanisms. Likewise, they identified 11 significantly mutated genes and one regulatory region largely converging on NF-kB signaling (76, 77) (**Table 2**). NF-kB pathway activation can occur through the overexpression of LMP1 or *via* inactivating mutations of NF-kB negative regulators, including CYLD, TRAF3, NFKBIA, and NLRC5 (78, 79). Significant somatic aberrations detected in HLA-A and NLRC5 (a transcription factor of MHC Class I genes) result in impaired antigen presentation, aiding in immune escape.

Chromosomal instability (CIN) is also a hallmark of NPC tumours. Copy number gains in multiple chromosomes including 1q, 3q, 7q, 8q, 12p, and 12q were frequently detected in NPC (60, 76). Homozygous deletion of 9p21.3 (CDKN2A/CDKN2B) as the most frequently altered chromosome region in a WGS study while Cyclin D1 (11q13) and MYC (8q24) have also been observed to be frequently amplified (76) (**Table 2**).

Global hypermethylation is a distinct feature in NPC and EBVaGC, particularly at CpG islands, leading to a characteristic CpG methylator phenotype (CIMP) associated with silencing of key tumour suppressor genes, including RASSF1, CDKN2A, and PTEN (80, 81). CDKN2A (coding for p16-INK4a protein that is key in cell cycle arrest) is one of the important TSGs in the cell cycle to be silenced through hypermethylation in both EBVaGC and NPC, and is considered an early event in tumourigensis of NPC. EBV latent proteins, such as LMP1 (in NPC) and LMP2A (in EBVaGC), have been shown to enhance the expression of DNA methyltransferase (DNMT) enzymes, resulting in the hypermethylated phenotype (82–85). Apart from CpG island methylation, histone modification especially elevated levels of H3K27me3, has been observed in NPC tumours and correlates with advanced disease and poor prognosis (86). Consistent with increased H3K27me3, elevated expression of EZH2 has been observed in NPC, repressing the activity of tumour suppressor genes including E-cadherin and p16 (87, 88).

The somatic molecular landscape in LELC is thought to be similar to NPC, demonstrating a similar spectrum of mutations in the NF-kB pathway in a cohort of 91 cases (64). Like NPC, PLELC was found to have aberrations in TP53, NF-kB, JAK/STAT, and cell cycle genes such as CDKN2A and CCND1 (64). A smaller study (*n* = 8) suggested that mutations in chromatin modification and cellular differentiation were less commonly observed in LELC compared to NPC (65). PLELC display few classical mutations identified in non-small cell or squamous cell cancer of the lung, with the exception of occasional KRAS and ERBB2 mutations (89). PLELC was also observed to have frequent copy number gains in chromosomes 5p, 12p, and 12q; copy number losses in chromosomes 3p, 5q, 13q, 14q, and 16q; and deletions in negative regulators of the NF-kB pathway (**Table 2**). Significantly amplified chromosome regions include 7p11.2, 9p24.1, 11q13.3 (also observed in LEL-HCC), and 12p13.2, while significantly deleted regions include 9p21.3 (CDKN2A/B), which is also commonly deleted in NPC.

Methylation of tumour suppressor genes (APC, PTEN, and RASSF1A) and cell adhesion molecules (THBS1 and E-cadherin) have been recognised in methylation studies performed for EBVaGC (90–93). Interferon regulatory factor 5 (IRF5), which mediates virus-induced innate immune responses *via* toll-like receptors (TLRs), has also been observed to be hypermethylated in EBV-positive gastric cancer cell lines and EBVaGC tissues (94). Recently, multi-omic analysis of gastric adenocarcinomas confirmed that EBV-positive gastric tumours are a molecular subtype of their own, with a highly distinct hypermethylated profile (95). Strikingly, almost all EBVaGCs demonstrate CDKN2A methylation. Compared to other gastric cancer subtypes (microsatellite instability, genomically stable and chromosomal instability), EBVaGCs have the highest frequency of PIK3CA mutations (80%), while TP53 mutations are rare. Unlike NPC and LELC tumours, mutations in the NF-kB pathway are uncommon in EBVaGCs.

The hypermethylated profiles observed in EBVaGCs may be a result of DNMT3b overexpression after EBV infection (96), as well as LMP2A-induced STAT3 phosphorylation resulting in increased expression of DNMT1 (84). In gastric cancer cell lines, DNA methylation appears to occur in tandem with histone modifications after EBV infection, with DNA methylation-sensitive genes correlating with decrease in active histone marks, including H3K4me2 and H3K27ac (97). In contrast to NPC, H3K27me3 repressive marks appear to be reduced in EBV-infected gastric cancer cell lines; however, regions with reduced H3K27me3 instead demonstrated elevated DNA methylation and maintained an inactive state (98).

In contrast to TP53 mutations being one of the most common mutations in NPC and LELC (64, 73), TP53 mutations are rare in EBVaGC. Early studies have suggested that TP53 is overexpressed in EBVaGC compared to EBV-negative gastric cancer (99, 100); however, other studies have also demonstrated the reverse (101, 102). Nonetheless, the absence of TP53 mutations in EBVaGC may partially explain the improved prognosis of EBVaGC compared to EBV-negative gastric cancers (103), as TP53 mutations are known to influence sensitivity to chemotherapy and radiation compared to EBV-negative gastric cancer (104).

## Tumour Immune Microenvironment of EBV-Driven Epithelial Cancers

Despite an expanding knowledge of the pathogenesis and somatic mutational landscape of EBV-driven epithelial cancers, there has been a lack of clinically effective novel therapies until the recent emergence of immune checkpoint inhibitors. The TIME of EBV-driven epithelial cancers comprises predominantly immune cells, some non-immune stromal cells including fibroblasts and non-cellular (extracellular matrix) components, as well as mediators such as cytokines, chemokines, and exosomes. A common feature of EBV-driven epithelial cancers is an immune-cell-rich but immunosuppressive stroma and the presence of immunogenic viral oncoproteins—a natural site and “soil” to identify potential therapies and biomarkers, as well as to further understand resistance mechanisms.

## Shaping of the NPC TIME by EBV Oncoproteins and Tumour-Mediated Recruitment

NPC at first glance appears to be an archetypical immune “hot” tumour with abundant immune infiltrate, especially T cells, in stroma (105). As compared to non-malignant nasopharyngeal tissues, which are B cell rich, NPC tumours have predominantly T cells, NK cells, myeloid-derived cells, and fibroblast infiltration (106). Compared to EBV-negative NPC tumours, the immune-suppressive T regulatory cells (Tregs), CD68+ myeloid cells, and exhausted CD8+ T cell subtypes are found enriched in EBV-positive NPC tumours (107). However, significant heterogeneity can be observed even among EBV-positive NPC tumours based on their immune profiles. Gene expression profiling from bulk tumours identified subtypes of NPC based on differences in proliferative signatures and the expression of immune genes (108). Importantly, tumours with a high proliferation but low tumour-infiltrating lymphocytes (TIL) signatures have the poorest prognosis.

**Figure 3:**
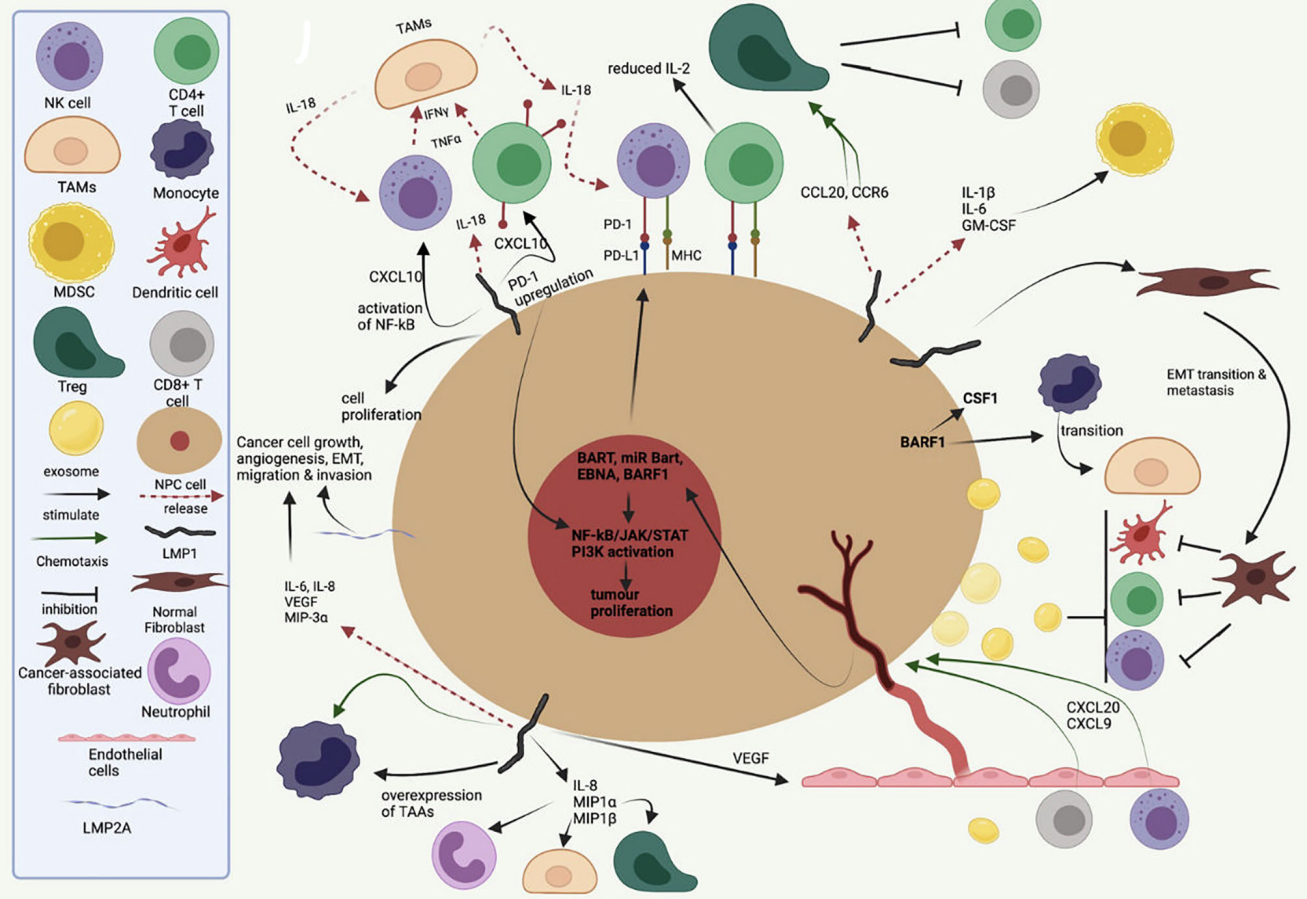
A symbolic representation of the role of EBV latent proteins and gene products in the tumour–immune cell cross-talk, recruitment of various immune cell into the tumour–stromal microenvironment and maintaining an immunosuppressive tumour immune microenvironment. Inspired by Lo AK-F et al. The Role of EBV-Encoded LMP1 in the NPC Tumor Microenvironment: From Function to Therapy. *Front Oncol.* 2021;11:640207. doi:10.3389/fonc.2021.640207.

The role of EBV latent genes, especially LMP1, in mediating immunosuppression in NPC, has been well recognised (**Figure 3**). Apart from its role in oncogenesis and metastasis, LMP1 modulates the TIME by the release of inflammatory cytokines [e.g., interleukin (IL)-6, IL-1α, IL-1β, IL-8, IL-10, interferon (IFN)-γ, and decreased IL-2] and chemoattractants [e.g., C-X-C motif chemokine ligand (CXCL)9, CXCL10, and CX3CL1] through the activation of NF-kB and STAT3, influencing immune evasion mechanisms and cell–cell interaction within the stroma (**Figure 3**) (25).

IFN-α and IFN-γ signaling pathways, key to anti-viral response, are activated in almost all the infiltrating immune cells in EBV-positive NPC, shown by an upregulation of IFN-induced genes, including ISG15, IFI6, IFI44L, IFIT3, and IFITM1 (106). Mouse models of B-cell lymphoproliferative disease also show marked upregulation of pro-inflammatory IFN-γ inducible chemokines after LMP1/LMP2A expression, including C-X-C chemokine receptor (CXCR)3 ligands such as CXCL9, CXCL10, and CXCL11 (109). In NPC, immunohistochemistry showed the presence of CXCL10-positive neoplastic cells in the majority of tumours, as well as CXCR3-positive lymphocytes in the accompanying stroma (110). This supports the idea that tumour-producing CXCL10 is able to recruit CXCR3+ infiltrating cells, including CD4 T cells, CD8 T cells, and NK cells. NPC cells also express cytokine and chemokine-encoding genes, including CX3CL1, CXCL10, C-C motif chemokine ligand (CCL)2, CSF1, IL-10, and Transforming growth factor beta 1 (TGF-β1). IL-10 is key in Treg maturation and response and contributes to an immunosuppressive TIME. LMP1 also upregulates IL-8, macrophage inflammatory protein (MIP)-1α, and MIP-1β, resulting the infiltration of multiple immune cells such as neutrophils, T cells, and macrophages (111).

Transfection of LMP-1 in Burkitt lymphoma cells promotes the expression of IL-18 (112),which is also overexpressed in NPC compared to normal nasopharynx. IL-18 is an inflammatory cytokine that induces IFN-γ production in T cells and NK cells. The increased IFN-γ production is thought to also activate CD68 macrophages to secrete IL-18, resulting in a positive feedback loop (25). IL-18 polymorphisms at the promoter region have also been associated with variable risk of nasopharyngeal carcinoma (113). NPC patients with elevated levels of CXCL10 have also been observed to have poorer prognosis (114), suggesting that despite their pro-inflammatory role, CXCL10 and IL-18 are unlikely to result in tumour-suppressive effects.

Overexpression of CCL20 has also been observed in NPC tumours and in the serum of NPC patients and found to be an unfavourable prognostic marker (115–118). Treatment of a humanised NPC mouse model with anti-CCL20 monoclonal antibody (mAb) resulted in a decrease in CD4+CD25+ regulatory T cells (Treg) recruitment (119). In addition, CCL20-containing exosomes from an NPC cell line could induce forkhead box protein P3 (FOXP3) expression in Tregs, which are critical for maintaining immune tolerance. Alternatively, NPC cell lines have also been observed to induce M2 macrophage polarisation, possibly through cytokines including TGF-β and IL-10 (120). Polarised M2 macrophages are themselves an important source of TGF-β, which promotes the induction of FOXP3+ Tregs from naïve precursors.

Apart from LMP1, other EBV viral oncoproteins and miRNAs are also implicated in the shaping of TIME. In NPC cell lines, EBNA1 activates TGFβ1-SMAD3 signaling and suppression of miR-200a, leading to increased CXCL12 expression and recruitment of CXCR4+ Tregs (121). EBNA1 also enhanced the production of CCL20, which is also important for Treg migration (122).Tumours with higher density of Treg infiltration correlate with EBNA1 expression and found to have a poorer survival. EBERs also induce inflammatory cytokines in NPC cells, *via* TLR3 and increased TNFα production (123, 124). Both EBER1 and EBER2 promotors have an NF-kB binding site, suggesting that EBERs and LMP1 (which activates both canonical and non-canonical NF-kB signalling) cooperate in an amplification loop to enhance the inflammatory response (123).

CD8+ T cells feature prominently in NPC stroma, but their functionality is disputed. In some studies, immunohistochemical staining for CD8+ TILs in NPC samples correlated positively with overall survival (125, 126), while other studies showed that high CD8+ TIL density was associated with poor disease outcome (127, 128). While the association of high CD8+ TIL density with a poorer prognosis may seem counter-intuitive, as CD8 is a marker of cytotoxic T-cells, it is important to note that CD8 also marks CD8+CD25+FOXP3+ regulatory T cells and IL-17-producing CD8+ T-cells (Tc17 cells), both of which have been observed in NPC tumours (129). The accumulation of CD8+CD25+FOXP3+ T cells (also known as CD8+ Tregs) has been observed in the tumour microenvironment of several cancers and are thought to suppress antitumour immunity. CD8+ Tregs are highly sensitive to IL-2 stimulation and suppress the proliferation of CD4+ T cells (130).

T-cell exhaustion is an acquired state of T-cell dysfunction characterised by a decrease in proliferation, cytokine production, and a reduction in cytotoxic capabilities. In NPC, T-cell exhaustion is thought to be a result of persistent antigen stimulation in the setting of chronic EBV infection. Programmed Death (PD)1, an inhibitory regulator of T cells and an accepted marker of T cell exhaustion, is upregulated in NPC compared to normal tissue. High PD1 expression within the tumour is associated with poorer disease control (131, 132).

Apart from its role in tumour inflammation discussed above, LMP1 also drives T-cell dysfunction through mitochondrial dysfunction and tumour exosomes. One study suggests the importance of hypoxia-related enrichment of miR-24 in NPC cells and NPC-derived exosomes, which then suppress the expression of MYC and FGF11 in TIL and decrease cellular oxidative phosphorylation level by disrupting MFN1-mediated mitochondrial fusion (133). The hypoxic microenvironment within NPC tumours also contributes to T-cell exhaustion, *via* upregulation of miR-24 and a reduction in mitochondrial mass (134). Hypoxia can also trigger upregulation of Programmed death ligand 1 (PDL1) in tumour cells in a hypoxia-inducible factor 1 alpha (HIF-1α)-dependent manner. Consistent with the above observations of T-cell exhaustion, EBNA1-specific CD8+ T cells from NPC patients have also been observed to be functionally defective in their response to EBV-infected cells (135).

Further characterisation has also shown the diversity of CD4+ T cell populations in NPC tumour. Regulatory CD4+CD25+FOXP3+ cells comprise 12% of all T cells (77). Compared with healthy controls, fewer circulating CD3+CD45RA+naïve (Tnaïve) and CD4+CD25- conventional T cells (Tconv) were found (107); a significantly higher percentage of CD25+ Treg cells from both the CD4+ and CD8+ lineages were found in PBMCs and TILs from NPC patients. Also, IL-17-producing CD8+ cells (Tc17) were detected at lower levels in NPC patients than in healthy controls (74, 107, 133). Tc17 cells were also lower in the peripheral blood, with most of the Tc17 cells being found among TILs. Interestingly, the majority of the Tc17 cells are derived from the CD45RO+ memory population (136) and may play a role in T-cell memory.

## Other Cell Populations in NPC TIME

A small number of natural killer (NK) cells (1%–9.5%), with varying amounts of other non-CD3+ cells can be found in the NPC stroma. B-cell populations are largely uncommon and are uninfected by EBV, suggesting that they were not clonally expanded but recruited to the TME through chemotaxis. There exist “double negative” (IGHD-/CD27-) B cells enriched in the NPC microenvironment and correlating to worse prognosis while other terminally differentiated B cells (plasma cells FCLR4+ memory cells) are associated with better prognosis. NK cells within NPC do not exhibit typical exhaustion markers but have cytotoxic signatures, suggesting possibly that they might not be significantly influenced by the immune-suppressive TIME (106). NK cells target virus-infected cells though germline-encoded receptors and NKG2A+ NK cells appear to be highly efficient at killing of EBV-infected B cells (137). They are also relevant in cytotoxic killing and tumour control—a recent scRNA study also identified key NK and T populations (138), which reported that NK markers were associated with better prognosis. Large amounts of dendritic cells (DCs) can be seen infiltrating tumour cells and mature DCs can be found mostly within the tumour cell area, with a few of them in the surrounding stroma (139). LAMP3+ DCs exhibit immunosuppressive properties and interact with exhausted T cells, Tregs, and proliferating T cells *via* CD28/B7 binding and IL-15 signaling in multiple tumours (140, 141). M1-polarised tumour-associated macrophages (TAMs) can be found in both tumour cell areas and in the stroma, while M2-polarised macrophages are often found in the stroma when present in high density (139). EBV infection has been shown to increase M2 TAMs polarization through NF-kB activation, leading to an increase in CD68+/CD206+ expression in a cell line study (78). Polarised M2 macrophages can also induce the conversion of naïve T cells into Tregs (122). Such TAMs exert immune-inhibitory effects and are also closely related to cancer-associated fibroblasts (CAFs) with a negative prognostic value (142). Tumour-associated neutrophils (TANs) are rarely studied but they can also be found in 10% of NPC stroma (122). LMP-1 mediated dysregulated glycolysis (through GLUT1), which in turn increases production of IL-1β, IL-6, and GM-CSF, and thus induce myeloid-derived suppressor cells (MDSCs) which are found to be important in T-cell suppression (143). MDSCs are elevated in the NPC TIME and are associated with worse prognosis and resistance to adoptive CTL therapy (144).

Fibroblasts are among the minority of cell population in NPC TIME but remain critical in their role in maintaining tumour invasiveness and also a immunosuppressive microenvironment. Fibroblasts in the NPC microenvironment can maintain the ECM and also secrete varied growth factors, including EGF, FGF, IGF1, TGF-β, and CSF, the latter mediating M2 TAM transition (145). CAFs have been associated with EMT transition and metastasis (1, 11, 146), and can be found around tumour cell nests, with varying amounts in different NPCs (138, 139). Again, LMP1 has been shown *in vitro* to facilitate transition normal fibroblasts (NF) to CAFs (121, 147). Alpha smooth muscle actin (αSMA), an immune marker for CAFs, was found to be correlated with CD34, an indicator of neoangiogenesis (121, 147). SDF-1/CXCL12, for which CXCR4 is the cognate receptor for (2), can be secreted by CAFs in various cancers to promote the growth of stromal fibroblasts. NPC usually sees an upregulation of CXCR4, thereby promoting tumourigenesis.

## Characterisation of TIME Through Transcriptomic Data

Bulk transcriptomic and recently single-cell RNA sequencing (scRNA-seq) also revealed different cell populations and subtypes of TIME in NPC—broadly categorised into inflamed, hot, and immune excluded. A few studies have separately identified subtypes of NPC according to immune and non-immune gene signatures, observing the immune gene signature-rich subtypes correlating with better survival outcomes (108, 148). Chen et al. described an immune-enriched stroma in 38% (43/113) of patients, characterised by significant enrichment of immune response signatures. The remaining patients were classified as a non-Immune Subtype (non-IS), which exhibited increased cell cycling. Within the immune-enriched subtype, a group of tumours (18/43 tumours, 42%) had activated stromal response signatures, including TGF-β associated extracellular matrix processes. This group of tumours also showed an increased CD8+ T-cell exhaustion signature and reduced survival compared to immune-enriched tumours without activated stromal response, showing a differing stroma–immune cell interaction.

Single-cell RNA sequencing and TCR repertoire sequencing analysis from more than 170,000 cells from 10 NPC tumours and matching PBMCs revealed 53 cell subtypes including tumour-infiltrating CD8+ T cells, Treg, and DCs, as well as malignant cells with different Epstein–Barr virus infection status. Trajectory analyses also revealed exhausted CD8+ T cells and immune-suppressive TNFRSF4+ Treg cells in tumours that might have been derived from peripheral CX3CR1+CD8+ T and naïve Treg cells, respectively. The authors also identified immune-regulatory and tolerogenic LAMP3+ DCs and noted intensive inter-cell interactions among LAMP3+ DCs, Treg, exhausted CD8+ T cells, and malignant cells, suggesting potential cross-talk to foster an immune-suppressive niche for the TIME (149).

## Immune Evasion Strategies by NPC

As mentioned earlier, on top the viral-mediated immunosuppressive milieu, NPC tumours also display a network of immune-escape mechanisms. NF-kB regulates multiple chemokines and immunosuppressive cytokines such as CXCL9, CXCL10, CX3CL1, and CCL20. Fifteen percent of primary NPC tumours downregulate HLA-class I protein (102) and 30% of EBV-associated NPC have somatic MHC class I gene aberrations (74). TAMs within NPC have been found to express indoleamine 2,3-dixoygenase (IDO), which can facilitate immune escape by impairing the cytotoxic action of T cells (150). Impaired secretion of IFN-γ and perforin by dysfunctional CD8+ T cells also leads to an inefficient antiviral and antitumour response (151, 152). Furthermore, IL-10 in NPC TIME downregulates MHC class II protein on antigen-presenting cells, inhibiting the activity of CD8+ T cells and IL-2 production from T helper cells (3). NPC tumour cells may also express a high level of CD40, which can bind to CD40L on infiltrating T cells, thereby preventing activation-induced cell death (AICD) (153). Separately, viral oncoprotein LMP1 was also found to upregulate PD1 on T cells, supporting T-cell anergy (151). NF-kB activation may also cause an LMP1-mediated induction of PDL1 in NPC cells, leading to immune escape (151). Lymphocyte activation gene 3 (LAG-3) and Hepatitis A virus cellular receptor 2 (HAVCR2) were also observed to be key immune checkpoint molecules in dysfunctional CD8+ T cells (138) while Cytotoxic T-lymphocyte-associated protein 4 (CTLA4+) FOXP3+ Tregs enrichment in EBV-positive NPC also highlights other immune checkpoint pathways. Furthermore, the Galectin-9/TIM-3 axis has also been reported to be one of the NPC-specific immunosuppression pathways (154).

The heterogeneity observed in the TIME plays out in treatment outcomes in a multicenter clinical trial for PD1 blockade in recurrent/metastatic NPC, with a 20.5% response rate to nivolumab (155). In this clinical trial, however, there was no statistical difference in overall survival between patients with PDL1-negative and PDL1-positive tumours (>1% expression). Interestingly, and counterintuitively, tumours with loss of HLA-A and/or HLA-B expression demonstrated improved survival in this clinical trial, suggesting that treatment response to PD1 blockade is a complex process that goes beyond antigen presentation on MHC Class I and inhibiting tumour PDL1 interactions.

## TIME Characterisation of LELC

There is a relative paucity of data on the TIME characterisation of LELC. Histologically, LELC is similar to NPC in that it has undifferentiated epithelial cells with prominent lymphoid infiltrate (156). Most of the TILs were found in the resting state and were mostly CD8+ and T-cell intracytoplasmic antigen (TIA-1) positive (26) and granzyme-B negative (157) with most of the CD8+ lymphocytes in the tumour cell nests and the surrounding stroma (158). TAMs can be found in larger quantities and closer to monocyte chemoattractant protein-1 (MCP-1) expressing tumour cells in PLELC than in other NSCLC (159) and are associated with a poorer prognosis. PLELC also exhibits several immune escape mechanisms. For instance, CD274 gene amplification is found to be associated with PDL1 upregulation (64). There is also a statistically significant upregulation of Bcl-2 in PLELC as compared to other types of non-small cell lung carcinoma (160). However, due to the rarity of the disease, very few studies involve the characterisation of the TIME and predictive biomarkers of PDL1, but case reports have suggested positive PDL1 staining, though one study noted that though LELC tumour cells expressed abundant PDL1, tumour‐specific CD8+ TILs mostly did not express PD1 (161).

## TIME Characterisation of EBVaGC

Compared with non-EBVaGC, EBVaGC has higher mRNA expression profiles of immune-related genes (IRGs), including Tregs and immune suppression checkpoint gene expression (162). EBVaGC also has an upregulation of IL-1β and IFN-γ, and lower tumour regulatory genes when compared to EBV negative GC (163). Expression levels of genes involved in antigen presentation are also significantly upregulated in EBVaGC, especially the genes that are involved in MHC class II presentation, regulation and T cell co-stimulation and survival (164). EBVaGC and NPC have not been compared directly in the way of transcriptomic signatures; however they both appear to have raised immune-related gene signatures, especially IFN-γ pathway genes.

Notably, in EBVaGC, the TME component is larger than in EBV-negative GC (165). EBVaGC was found to have a larger number of CD3+ T lymphocytes and CD68+ macrophages than gastric cancers that are EBV negative. One study noted that higher CD3+ T lymphocyte density is associated with an improved 5-year overall survival (20). The ratio of CD8+ to CD4+ infiltrating T cells is often 10:1 (139). Increased expression of IL-1β allows for the recruitment of lymphocytes to prevent direct contact between EBV-associated cytotoxic T cells and the tumour cells, thereby inhibiting apoptosis. Upregulation of IFN-γ allows for increased expression of IDO-1. EBVaGC also exhibits several immune escape mechanisms. They are found to have an upregulation of PDL1, PDL2 loci (166), and IDO-1 (92). Another study showed that EBVaGC tumour cells generally express high levels of PDL1 and also inhibit T-cell proliferation through upregulation of IFN-γ (167). Also, increased expression of CCL22 in EBVaGC can attract more Tregs (168).

One study reports that EBVaGC patients with higher PDL1 expression have better prognosis (169). Recently, a Korean study again showed PDL1 status to correlate with EBV status in a large case series of GCs (170). Another study reported that EBV tumours were most infiltrated with CD8+ CTL and macrophages (28% and 22% of all intratumoural cells, respectively), with an intermediate frequency of CD4+ T cells (20%) and low rate of Tregs (4%) (171). A high density of CD204+ TAMs has been associated with the aggressive GC tumour behaviour and worse survival of GC patients. Low density of CD204+ TAMs is associated with EBV infection, which may explain the favourable outcome of EBV-associated gastric carcinoma (168). The number of DCs in EBVaGC is also higher than that in non-EBVaGC (172). The density of neutrophils in the EBVaGC tumour microenvironment is markedly low. In some cases, neutrophils are not detected in the tumour (173).

Despite a lack of comparative data, immune checkpoint upregulation, especially PD1/PDL1, appears to be a feature of EBV-driven epithelial cancers, and immune cells are overall abundant in the stroma of these cancers, though with varying frequencies. The viral presence, in particular the role of LMP1 and MiR-BARTs in NPC, heavily influences the shaping of the TIME and a dysfunctional CTL state. In particular, the immunosuppressive Tregs and myeloid lineage cells such as MDSCs and TAMs are common culprits in all three EBV-driven epithelial cancers. As mentioned, EBV miRNAs can also reduce immune surveillance and attenuate T-cell-mediated immune control in EBV-associated diseases (174).

## Future Directions and Novel Therapeutics

Our centre is currently comparatively characterising the tumour immune stroma of NPC and pulmonary LELC through an integrated multispectral imaging technique that simultaneously detects viral RNA and immune cell protein markers in formalin-fixed, paraffin-embedded (FFPE) tissues of EBV-associated epithelial cancers (**Figure 2**) (175). We hope to better understand the immune cell phenotypes and other biomarkers in the tumour immune microenvironment. However, despite the best efforts to characterise the treatment-naïve TIME, the immune milieu of solid tumours, while influencing drug response, is in turn affected by cytotoxic chemotherapy and other therapeutic approaches (176). Systemic therapy does not only exert a selection pressure on cancer cells, but also alters the TIME during the course of treatment. Hence, a temporal evolution of the TIME may eventually be required to understand the disease and treatment trajectory, which could require multiple invasive tumour biopsies, or this could be indirectly obtained through PBMCs, circulating DNA or tumour exosomes and perhaps future novel strategies such as functional imaging.

A deeper understanding of the key signaling/molecular pathways will contribute towards rational drug combination and drug development in EBV-associated cancers. With an understanding of the molecular pathways commonly subverted in the tumourigenesis, potential therapeutic focus including overcoming metabolic reprogramming by targeting glycolysis enzymes, epigenetic regulators, and other specific oncogenic pathway inhibitors may be envisioned; however, they remain in the nascent stages of drug development and translational research (177). For example, in EBV-associated NPC, some clinically important mutations with potential targeted therapies including PIK3CA, EGFR, FGFR1, and BRCA/ATM are uncommon; fusion genes such as FGFR3 are also uncommon. Noting this, perhaps the key characteristic of EBV-driven epithelial cancer—its significant EBV gene product and immune cell landscape—may be a main area of research into novel therapy.

The mainstay of treatment for early-stage NPC is radiation therapy (RT), with the addition of chemotherapy for more locally advanced disease. For metastatic disease, chemotherapeutic approach (e.g., gemcitabine plus cisplatin/carboplatin, taxanes, and fluoropyrimidines) is the current standard of care though with limited efficacy. Molecular targeted therapies such as EGFR inhibitors and VEGF inhibitors have shown limited efficacy and have not been adopted in standard treatment currently (178). Recently, a collaborative study involving our centre has shown that upregulation of somatostatin receptor 2 (SSTR2) in NPC is also mediated by LMP1, and this may potentially be amenable to therapeutic blockade (179).

For early-stage LELC disease, radical surgical resection is usually performed for potentially curative intent. Systemic chemotherapeutic agents, similar to those used in NPC, have been the main treatment option for metastatic disease, but with limited data owing to the rarity of this type of cancer. Case series of PLELC also report a high response rate to ICI, where 8 out of 10 patients (80%) responded with a median PFS of 15 months, significantly higher than that of a matched cohort of patients on chemotherapy (180).

Treatment modalities of early EBVaGC involve a surgical approach such as endoscopic mucosal resection or gastrectomy. Locally advanced EBVaGC is treated similarly as for other types of GCs, including peri-operative chemotherapy with primary resection. For advanced disease, a palliative approach is usually taken, which may involve cytotoxic chemotherapy (fluoropyramidines, platinums, taxanes, and irinotecan) and VEGF-R antagonist ramucirumab to treat the disease systemically, though PFS and overall survival are longer than that of non-EBVaGCs (45). Interestingly, immune checkpoint inhibitors such as pembrolizumab may have superior efficacy in EBVaGC (61) as one study showed 100% (6/6) response rate of EBVaGC to pembrolizumab, but larger studies are needed to validate this finding.

ICIs such as pembrolizumab and nivolumab have shown efficacy both as single agents and in combination with chemotherapy in advanced GC. With PDL1 as a positive predictive biomarker, it has been shown that in subgroups of patients with raised PDL1 combined positive score (CPS) or tumour proportion score (TPS), ICI and chemotherapy combination outperforms chemotherapy in disease control and progression-free survival (PFS) (178). Though there is an absence of clinical trials in EBVaGC, high disease responses to ICI in a few case series have been reported. Overall, this bears a striking resemblance to the recently presented JUPITER-2 study, which showed chemotherapy and ICI outperforming chemotherapy alone in advanced NPC.

There appears to be plasticity in the exhausted TILs and strategies to re-activate these dysfunctional cells including employing anti-PD1 and PDL1 blockade therapy, namely, pembrolizumab, nivolumab, camrelizumab, toliparimab, and anti-CTLA1 therapy ipilimumab. Clinical trials using immune checkpoint inhibitors targeting the TIME interactions have shown some promises in metastatic NPC. The KEYNOTE-028 Phase 1b clinical trial of pembrolizumab single agent in PDL1 positive chemo-refractory NPC patients showed a response rate of 26% (7 of 27 patients) (178), which is comparable to the single agent response rate of pembrolizumab in other solid organ tumours (181). This was followed by the NCI-9742 Phase 2 clinical trial demonstrating a response rate of 20.5% (9 of 45 patients). Biological markers for treatment response to anti-PD1 therapies have focused largely on the presence or absence of PD1/PDL1 on immunohistochemistry (182). More encouraging results emerged recently—a combination of chemotherapy and anti-PD1 Toliparimab (JUPITER-2) in the first-line setting of metastatic NPC yielded improved PFS of 11.7 months vs. 8 months in standard of care chemotherapy, hazard ratio (HR) 0.52, 95% CI [0.36, 0.74]; *p* = 0.0003 (ASCO abstract 2021). This highly encouraging result showing efficacy of combining ICI with cytotoxic chemotherapy in NPC may also be applied in other EBV-driven epithelial cancers.

We await the updated outcomes of combination anti-PDL1 and anti-CTLA4 antibody in the first-line treatment of advanced NPC (NCT03097939) though early outcomes suggest a 14/40 (35%) response rate and a median duration of response of 5.9 months (ESMO Asia 2020). In the same vein, combination PD1 and CTLA4 therapy may be extended to other EBV-associated tumours that have significant populations of Treg and APCs expressing CTLA4. In light of the viral-related immune escape mechanisms and T-cell exhaustion within the TIME, combining immune checkpoint inhibitors can be further explored, including LAG3, TIM3, HAVCR2, and TIGIT inhibitors, among other novel combination therapies, to further “rescue” or “re-activate” the dysfunctional TILs. Multiple LAG3 (MK-4280, TSR-033, and IMP321) antibodies are often evaluated in combination with anti-PD1; anti-HAVCR2 and anti-TIGIT antibodies are in the early-phase development.

The lack of expected biomarkers to predict treatment response to anti-PD1 treatment was observed in the NCI-9742 nivolumab study for recurrent/metastatic NPC, where there was no statistical correlation with tumour PDL1 expression (155, 167). Surprisingly, patients with loss of HLA-A and/or HLA-B in their tumours had improved PFS compared to tumours expressing both HLA-A and HLA-B (1-year PFS 30.9% median PFS 4.8 months, versus 1-year PFS 5.6%, median PFS 1.8 months). This is paradoxical because recognition by the T-cell receptor of peptides presented on MHC-I is essential for cytotoxicity, yet tumours with intact MHC-I appear to have a poorer prognosis. Wang et al. highlighted the importance of immune status in prognostication—PDL1 and B7-H4 on tumour cells and PDL1, B7-H3, B7-H4, IDO-1, VISTA, ICOS, and OX40 on intralesional immune cells (183). Gene signatures of macrophages, plasmacytoid DCs, CLEC9A+ DCs, natural killer cells, and plasma cells were also associated with improved PFS (138). We anticipate that the identification of immune cell signatures will better prognosticate and stratify EBV-associated cancers to streamline and select for more optimal immunotherapies.

Altering the tumour microenvironment through adoptive T or NK cell infusion, or through the activation of new tumour antigens *via* rational combination with radiation therapy or specific chemotherapeutic agents, may also “switch” the tumours from “cold” to “hot”, allowing better T-cell infiltration and tumour killing. Combining therapies with anti-angiogenic therapy in specific tumours with a strong presence of pro-angiogenic signatures and fibroblasts may also improve immunotherapy efficacy by improving T-cell infiltration in the stroma. Multiple studies have looked into combining bevacizumab with chemotherapy or with anti-PD1 therapy (NCT03813394) (184).

Outside of ICI, cancer vaccines (185) and adoptive T-cell therapy have also been actively investigated in EBV-driven cancers, mainly in NPC and EBV-associated lymphoid malignancies. However, identifying immunogenic EBV oncotargets remain challenging. Cell therapy and vaccine targeting EBNA1, LMP1/2, BARF1, and induction of EBV lytic cycle genes have been attempted for NPC. Despite LMP1 and LMP2 being *bona fide* targets, their general low immunogenicity and variable expression pose as potential barriers. Potential targets for future cell therapy and drug development could include other EBV viral products such as microRNA family, BARF1, and lytic enzymes (186). Universal off-the shelf EBV-specific T-cell therapy (EBVST) may be more conveniently delivered against EBV-associated epithelial cancers, which could reduce the vein-to-vein time from start of production of EBV targeting T cells to delivery into patients. NK cell therapy, CAR-T cell therapy, and TIL infusion early-phase trials in EBV-associated epithelial cancers are ongoing, with NK cell therapy showing some early signals of safety and feasibility (187).

Our centre reported the early phase study of adenovirus transfected LMP1 and LMP2 into DC as a cell-based vaccine in advanced pre-treated, chemo-refractory NPC patients but this yielded very modest objective clinical efficacy. Other studies of cancer vaccines and adoptive T-cell therapy in the form of CTL and NK cell infusion have both been investigated in the setting of Phase I/II trials, with some promising early signals (188). Of note, one meta-analysis on CTL trials in NPC demonstrated a reasonable outcome of PFS and tumour control (189). A randomised phase III study comparing up to six infusions of EBV-specific CTL following induction gemcitabine and carboplatin versus gemcitabine-carboplatin in stage IV recurrent or metastatic NPC in the first-line setting has completed accrual of its 330 patients and awaits final survival analysis, following the encouraging results achieved in the phase II trial of the same chemotherapy and then sequential EBV-specific CTL regimen at our centre (190).

We read with interest a recent case report of a metastatic NPC patient who had complete response to PD1 blockade following EBVST (191). In our centre, we treated one patient with advanced PLELC who had previously received multiple lines of chemotherapy and anti-PD1 therapies, with serial multiple infusions of EBVST following gemcitabine-cisplatin induction. She achieved an initial significant reduction in EBV DNA titer as well as subsequent metabolic remission on Positron Emission Tomography (PET)-Computed Tomography (CT) imaging when radiation therapy was introduced for local disease control even as she had multiple lung and lymph node metastases (unpublished). We also note the efficacy of EBVST against EBVaGC cell lines (192). The future will likely trend towards combination and sequential immunotherapies such as adoptive cell therapy or therapeutic cancer vaccination with immune checkpoint inhibitor.

Other novel immune-based strategies remain investigational. A recent study showed that LMP1 signaling in B cells led to an overexpression of tumour associated antigens presented on MHC-I and II, and the upregulation of costimulatory ligands CD70 and OX40L, thereby inducing potent cytotoxic CD4+ and CD8+ T-cell responses (193). Such an approach may be potentially exploited in NPC by *ex vivo* overexpression of LMP1 in tumour cells, with the goal of increasing tumour antigen presentation to prime autologous cytotoxic T cells. Anti-CD70 therapy is also currently in early-phase studies and we await the results of combination therapies with chemotherapy or with anti-PD1/PDL1 therapy (194). B-cell directed therapy with a focus on plasma and memory B-cell pool, which are associated with better clinical outcomes (195), are preliminary but may result in clinical efficacy against EBV-associated cancers. Also, targeting suppressive immune cells such as MDSCs, TAMs, Tregs, and DCs through specific mediators and chemokine antagonists have been tested against other cancers and would be rational against NPC to potentially unleash an anti-tumour immunity.

## Conclusion

While clinically distinct, NPC and LELC share similarities in genomic mutational landscape, but much is still unknown about how their transcriptomic landscapes and tumour microenvironments differ. A deeper cumulative understanding of the commonalities across all the EBV epithelial cancers and their surrounding TIME, as well as their distinct differences, will help better design therapies that can be used across these cancers as well as drugs that are more specific for each respective EBV driven cancer type. This is more possible than ever before by harnessing the new science that enables much deeper examination at the single-cell level, at the metabolome, epigenome, proteome, immunome, and beyond.

## Author Contributions

SH and JJT contributed equally to manuscript writing and share first authorship. AC and CL contributed to manuscript writing and illustration. JY and CML contributed to images of data. HCT is the corresponding author and also contributed to conception, guidance, and manuscript writing. All authors contributed to the article and approved the submitted version.

## Funding

This work was funded by Large Collaborative Grant, National Medical Research Council, Singapore (HCT) and Transition Award TA20nov-0025, National Medical Research Council, Singapore (JKT).

## Conflict of Interest

The authors declare that the research was conducted in the absence of any commercial or financial relationships that could be construed as a potential conflict of interest.

## Publisher’s Note

All claims expressed in this article are solely those of the authors and do not necessarily represent those of their affiliated organizations, or those of the publisher, the editors and the reviewers. Any product that may be evaluated in this article, or claim that may be made by its manufacturer, is not guaranteed or endorsed by the publisher.
